# Immune responses and immunotherapeutic approaches in the treatment against cancer

**DOI:** 10.1007/s10585-024-10300-7

**Published:** 2024-08-18

**Authors:** Stanley P Leong

**Affiliations:** California Pacific Medical Center and Research Institute, University of California School of Medicine, San Francisco, USA

**Keywords:** Immune Responses and Immunotherapy against Cancer

## Abstract

Cancer cells within a population are heterogeneous due to genomic mutations or epigenetic changes. The immune response to cancer especially the T cell repertoire within the cancer microenvionment is important to the control and growth of cancer cells. When a cancer clone breaks through the surveillance of the immune system, it wins the battle to overcome the host’s immune system. In this review, the complicated profile of the cancer microenvironment is emphasized. The molecular evidence of immune responses to cancer has been recently established. Based on these molecular mechanisms of immune interactions with cancer, clinical trials based on checkpoint inhibition therapy against CTLA-4 and/or PD-1 versus PD-L1 have been successful in the treatment of melanoma, lung cancer and other types of cancer. The diversity of the T cell repertoire is described and the tumor infiltrating lymphocytes within the cancer may be expanded ex vivo and infused back to the patient as a treatment modality for adoptive immunotherapy.

## Introduction

Cancer is a heterogeneous disease arising from genomic mutations [1] or epigenetic changes [2]. Within a cancer cell population, heterogeneous clones may develop resulting in cancer heterogeneity within a tumor, between different clones, metastatic deposits and patients. The cancer microenvironment exerts a selective force akin to Darwinian “natural selection” [3–5] that promotes the development of invasive clones to metastasize from the primary site to distant sites, in accordance with Paget’s seed and soil hypothesis [6] for cancer spread. Cancer metastasis may occur through lymphatic vessels to the sentinel lymph nodes or through blood vessels, or via both pathways [7, 8]. The molecular mechanisms of cancer spread are under intense study. While in the literature, the genetics [9] and proteomics [10] of cancer have been emphasized, the cancer microenvironment, especially the immune response to cancer and the T cell repertoire of the host has not been highlighted. In this review, we want to review the immune responses relating to the T cell repertoire to cancer. It is important to understand the dynamic interactions between cancer metastasis and T cell repertoire. Perhaps these interactions are different during different stages of cancer evolution from a single cell to a clonal population with development of invasive clones to metastatic sites.

A review of the discovery of T cells over the past 60 years has been well documented by Biolegend [11]. T cell milestone discoveries include the thymic function in the 1959s, as the thymus has been found to be the site of T cell development [12]. CD8 + T cells were discovered in 1975, when they were depleted by antiserum, their cell-mediated cytotoxicity was abolished [13]. Kohler and Milstein developed monoclonal antibodies from hybridomas in 1975 [14] and won the Nobel Prize in Physiology or Medicine in 1984, jointly with Niels Jerne [15], Their discovery has boosted biological research significantly to design monoclonal antibodies against specific T cell targets [16]. In 1979, a monoclonal antibody, OKT4, was developed, which was able to sort out CD4 positive and negative populations. The positive fraction was identified as CD4 + helper T cells, being primarily responsible for adaptive immune response by producing cytokines to support other cells such as macrophages, B cells and CD8 cells. Thus, CD4 + helper T cells are responsible for facilitating many cellular and humoral immune responses against pathogens and cancer. Another subset, namely regulatory T cells (Tregs), causes immunosuppression in reducing immune responses. Resting T helper cells, being characterized by a few cell surface molecules such as CD4, CD45RA, CD62L, and CCR7, are known as naïve T helper cells, which circulate in the blood. They may be fully activated by antigen presenting cells such as dendritic cells, macrophages, and B cells, involving stimulatory signals through the T cell receptor and costimulatory signals through CD28. The negative population was cytotoxic [17]. In the late 1980s, Mosmman and Coffman identified surface markers on CD4 T helper cells namely Th1 and Th2 [18, 19]. Th1 cells are found to have anti-viral and antibacterial immunity, producing cytokines including IFN-γ, IL-2, and TNF-α. On the other hand, Th2 cells react against extracellular pathogens, generating IL-4, IL-5, and IL-13. In the three decades from 1990s to 2010s, multiple T helper subsets have been discovered including regulatory T cells and others with unique immune functions. Likewise, numerous subclasses of CD8 + T cells have been identified [11].

Due to the significance of CD4 + and CD8 + T cells in the immune response against cancer, further detailed description is included as follows:

CD4 + T cells, as recognized above as T helper cells, are essential white blood cells that orchestrate the body’s defense mechanisms against pathogens, playing pivotal roles in both adaptive and innate immunity. Originating from stem cells in the bone marrow and maturing in the thymus, these cells are central to ensuring the immune system’s self-tolerance and operational efficacy. Through encounters with antigens presented on antigen-presenting cells (APCs) via MHC II molecules, naive CD4 T cells are prompted to diversify into several subtypes, including Th1, Th2, Th17, Tfh, and Treg cells, each tailored to specific immune functions. CD4 + T cells enhance the immune response by aiding other immune cells such as CD8 T cells and B cells, through the secretion of cytokines which assist in their activation and development. Importantly, they play a regulatory role, preventing excessive immune reactions and autoimmunity, with Treg cells being crucial for maintaining immune tolerance. Following an infection, CD4 T cells can transition into memory cells, ensuring a swift and robust response upon subsequent exposures to the same pathogen, thus contributing to lasting immunity. Leveraging the regulatory and adaptive capabilities of CD4 + T cells forms the basis of various immunotherapeutic approaches, including vaccines and treatments for cancer and autoimmune conditions. In essence, CD4 + T cells are indispensable to immune system regulation and response, highlighting their importance in both health and disease.

CD8 + T cells, also known as cytotoxic T cells, are crucial immune cells responsible for detecting and destroying cells that are infected, malfunctioning, or cancerous. These cells develop from the same precursors as CD4 + T cells in the bone marrow and are characterized by their CD8 glycoprotein expression. They become specialized during their maturation in the thymus. CD8 + T cells identify infected or abnormal cells by recognizing peptides presented by MHC class I molecules on the surface of all nucleated cells. When activated, they can kill target cells by releasing substances like perforin and granzymes, which trigger apoptosis, and by activating Fas ligand, which promotes death through receptor pathways. In addition to their cytotoxic function, CD8 + T cells produce several cytokines, such as interferon-gamma (IFN-γ), which helps activate other immune components and strengthens the defense against microbes. Their activity is finely tuned through a mix of activating and inhibitory signals, ensuring they target only the appropriate cells while sparing healthy tissue. CD8 + T cells are also vital in fighting cancer, recognizing and eliminating cancer cells by identifying cancer-associated antigens. This capability places them at the forefront of cancer immunotherapy research, with treatments like checkpoint inhibitors and CAR-T cell therapies exploiting their targeting ability to combat various cancers. Moreover, CD8 + T cells play a significant role in developing immunological memory. After an initial immune response, a subset of these cells remains as memory T cells, which are primed to respond more vigorously and quickly if the same antigen is encountered again. This mechanism is fundamental to the success of many vaccines. Overall, CD8 + T cells are essential for the immune system’s capacity to identify and eliminate harmful cells, contributing to direct cell destruction, cytokine production, cancer control, and lasting immune memory. Their multifaceted roles are critical for health maintenance and disease prevention.

**Fig. 1:**
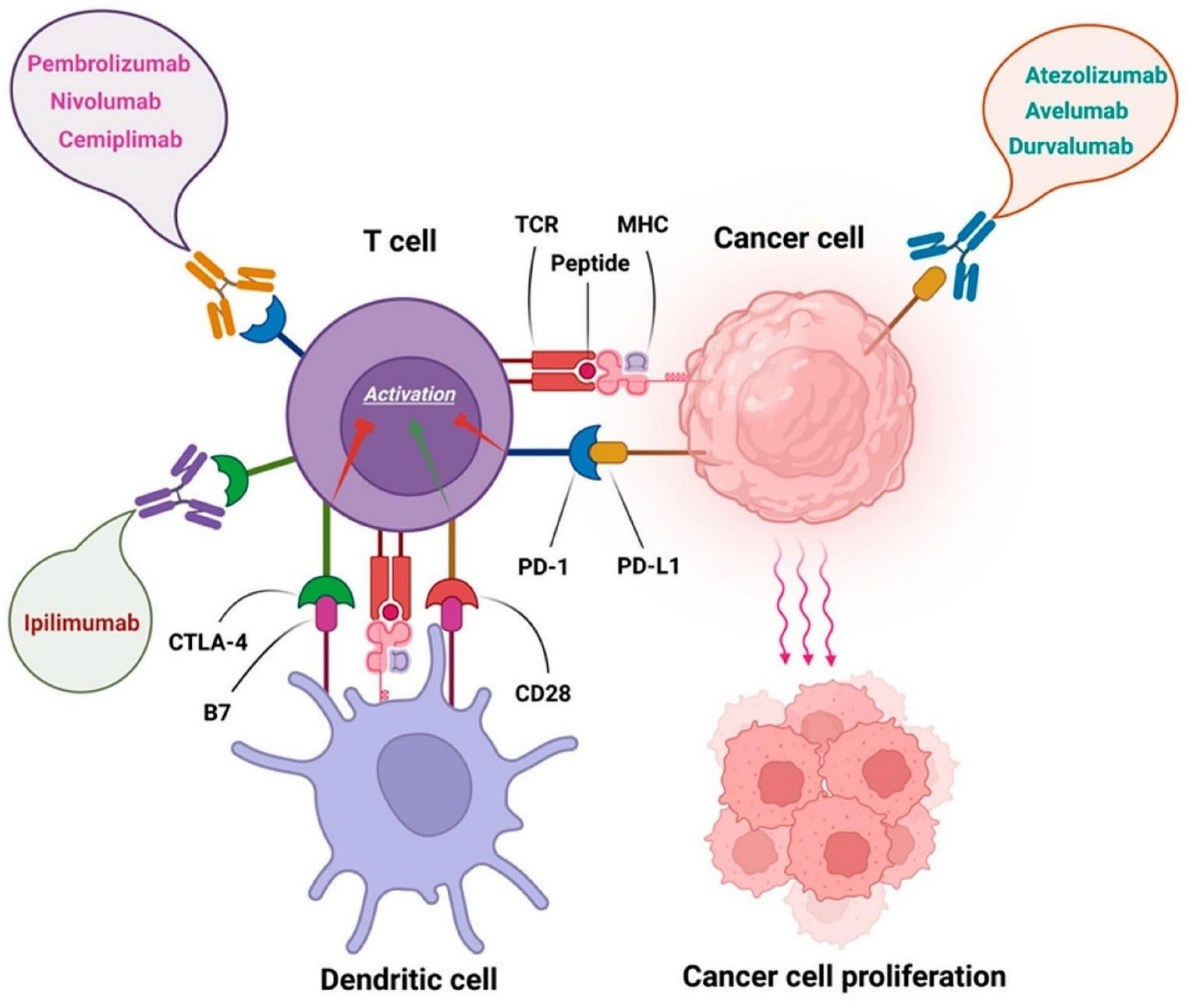
The exploitation of programmed cell death 1 (PD-1) and cytotoxic T lymphocyte antigen 4 (CTLA-4) by cancer cells represents a sophisticated mechanism of immune evasion. Both PD-1 and CTLA-4 are co-inhibitory molecules on the T cell surface to negatively regulate immune response by T cells to ensure that the immune system would not damage the body’s own tissues. However, these inhibitory mechanisms result in the dampening of their activity against the cancer. By expressing or inducing the expression of the ligands for PD-1 (PD-L1) and CTLA-4, cancers can effectively “turn off” T cells that might otherwise attack them, thus, resulting in cancer cell proliferation as shown in the figure. Immune checkpoint inhibitors as represented in the figure block the interactions between PD-1 and its ligands (with anti-PD-1 such as Pembrolizumab, Nivolumab and Cemiplimab and anti-PD-L1 therapies such as Atezolizumab, Avelumab and Durvalumab) or inhibiting CTLA-4 (with anti-CTLA-4 therapies such as Ipilimumab). Thus, these drugs, which have been approved by FDA, can reinvigorate T cells, resulting in the destruction of cancer cells [23]. Figure reprinted from Current Oncology through Creative Common CC BY license

The breakthrough in the molecular mechanisms of immune responses against cancer was the discovery of immune checkpoint receptors such as CTLA-4 and PD-1 being associated with T cell inhibition and cancer growth for which the Nobel Prize in Physiology of Medicine in 2018 was awarded to the joint discoverers, James Allison and Tasuko Honjo [20]. Thus, therapy by immune checkpoint blockade has resulted in a renewal of activated T cells, which may attack and destroy cancer [21, 22] (Fig. 1). While it is important to understand the molecular mechanisms of cancer proliferation and metastasis, it is also critical to understand how the extent and diversity of T cells within the cancer microenvironment are related to the blockade and activation against cancer. The T cell repertoire within the cancer microenvironment of the primary site versus the metastatic site should be considered as an ever-changing profile in relationship to cancer growth, which is associated with mutation and epigenetic changes as mentioned above with acquisition of neoantigens [24]. These changes result in cancer heterogeneity, which poses a challenge for the immune system to adjust and deal with different heterogeneous clones. When a cancer clone breaks through the surveillance of the immune system, it wins the battle to overcome the host.

## Clinical evidence of cancer immunity against cancer

### Summary of clinical trials using ipilimumab against metastatic melanoma based on checkpoint inhibition of CTLA-4

Schadendorf et al. [25] performed a meta-analysis of overall survival data from multicenter studies consisting of 10 prospective and two retrospective studies of 1,861 patients given ipilimumab, a monoclonal antibody against CTLA4 [25]. The overall survival rates showed a plateau at 21% beginning around year 3, which was independent of prior therapy or ipilimumab dose. These data support the evidence of long-term survival durability in ipilimumab-treated patients with advanced melanoma [25].

### Comparison of Ipilimumab alone, Ipilimumab plus gp 100 peptide vaccine and gp 100 peptide vaccine alone in the treatment of metastatic melanoma

In a prospective randomized study, a total of 676 HLA-A*0201–positive patients with unresectable stage III or IV melanoma, with disease progression during therapy for metastatic disease were randomized to receive ipilimumab (at a dose of 3 mg per kilogram of body weight) along with a gp100 peptide vaccine (403 patients), ipilimumab alone (137), or gp100 vaccine alone (136) in a 3:1:1 ratio. The study investigated the efficacy and safety of ipilimumabin treating patients with previously treated metastatic melanoma. The study found a significant improvement in overall survival among patients treated with ipilimumab. Patients receiving ipilimumab in combination with the gp100 peptide vaccine had a median overall survival of 10.0 months, while those receiving ipilimumab alone had a median overall survival of 10.1 months. In contrast, patients treated with the gp100 peptide vaccine alone had a median overall survival of only 6.4 months. The hazard ratio for death was significantly lower in the ipilimumab-treated groups compared to the gp100-alone group. For ipilimumab with gp100 vaccine, the hazard ratio was 0.68 (*P* < 0.001), and for ipilimumab alone, it was 0.66 (*P* = 0.003), indicating a significant reduction in the risk of death with ipilimumab treatment. No significant difference in overall survival was found when comparing the two ipilimumab treatment groups (ipilimumab plus gp100 vaccine vs. ipilimumab alone), with a hazard ratio of 1.04 (*P* = 0.76). These findings suggested that the addition of the gp100 vaccine to ipilimumab treatment did not significantly impact overall survival. Significant side effects were more common in patients receiving ipilimumab. However, most adverse events associated with ipilimumab treatment were reversible with appropriate management. The study concluded that ipilimumab, either alone or in combination with the gp100 peptide vaccine, significantly improved overall survival in patients with previously treated metastatic melanoma compared to treatment with gp100 vaccine alone. Despite the increased incidence of adverse events with ipilimumab, these were generally manageable.

This clinical trial highlights the potential benefits of immunotherapy with ipilimumab in metastatic melanoma. The findings support the use of ipilimumab as a valuable treatment option in the context of metastatic melanoma, particularly for patients who have received prior treatments [26].

### Summary of clinical trials using pembrolizumab against metastatic melanoma based on checkpoint inhibition of PD-1

Using anti-programmed-death-receptor-1 (PD-1) antibody, pembrolizumab, the efficacy and safety of pembrolizumab at doses of 2 mg/kg and 10 mg/kg every 3 weeks in advanced melanoma patients (aged ≥ 18 years) who failed ipilimumab treatment were studied in an open-label and multicenter phase 1 trial. Patients were randomly assigned a 1:1 final ratio of intravenous pembrolizumab at 2 mg/kg every 3 weeks or 10 mg/kg every 3 weeks until disease progression, significant toxicity or withdrawal of consent. The overall response rate was the end point according to the Response Evaluation Criteria In Solid Tumors (RECIST, version 1.1) by an independent review committee. In this study, 173 patients received pembrolizumab 2 mg/kg (*n* = 89) versus 10 mg/kg (*n* = 84) with a median follow-up of 8 months. The overall response rate was 26% at both doses (difference 0%, 95% CI − 14 to 13; *p* = 0.96). Safety profiles in the 2 mg/kg and 10 mg/kg groups were similar and no drug-related deaths were reported with the treatment being well tolerated. The authors indicated their results supported the conclusion that pembrolizumab at 2 mg/kg or 10 mg/kg every 3 weeks might be effective in patients who had failed ipilimumab treatment [27].

### Summary of clinical trials comparing pembrolizumab (against PD-1) and ipilimumab (against CTLA-4) against metastatic melanoma

The Keynote-006 randomized phase 3 study serves as a crucial evaluation comparing pembrolizumab, a PD-1 blocker, against ipilimumab, an inhibitor of the CTLA-4 immune checkpoint, in the treatment of advanced melanoma. The goal of the study was to determine the effectiveness and safety of pembrolizumab relative to ipilimumab, where 834 patients were evenly randomized across three groups to receive either pembrolizumab (at a dose of 10 mg per kilogram of body weight) at intervals of 2 or 3 weeks, or ipilimumab (at 3 mg per kilogram) every 3 weeks. The main objectives were to observe the duration of progression-free survival and overall survival rates.

Key findings from the study revealed that at the six-month interval, pembrolizumab significantly surpassed ipilimumab in progression-free survival rates, with 47.3% for the biweekly and 46.4% for the triweekly pembrolizumab groups, compared to 26.5% for ipilimumab. This demonstrated a substantial reduction in the risk of disease progression for patients receiving pembrolizumab. Furthermore, the one-year survival rates were markedly better in the pembrolizumab groups (74.1% for biweekly and 68.4% for triweekly dosing) versus the ipilimumab group (58.2%), signifying a considerable survival advantage for those treated with pembrolizumab.

Additionally, the study found a significantly higher treatment response rate in patients administered pembrolizumab (33.7% for biweekly and 32.9% for triweekly dosing) as opposed to those given ipilimumab (11.9%). Moreover, pembrolizumab treatments resulted in fewer severe adverse reactions, with serious treatment-related adverse events being less frequent in the pembrolizumab groups (13.3% and 10.1%) than in the ipilimumab group (19.9%).

Conclusively, the study underscores pembrolizumab’s superiority over ipilimumab in enhancing progression-free survival, overall survival, and response rates among patients with advanced melanoma. Pembrolizumab also exhibited a more favorable safety profile, characterized by a lower incidence of serious adverse effects. Notably, the efficacy of pembrolizumab remained consistent across its different dosing schedules, suggesting flexibility in treatment management.

These findings consisting of pembrolizumab’s efficacy and safety profile, have significantly contributed to the evolving landscape of treatment guidelines and clinical practices, offering a pathway for personalized treatment strategies that could improve patient adherence and enhance quality of life [28].

### Summary of clinical trial using pembrolizumab (against PD-1) and ipilimumab (against CTLA-4) against metastatic melanoma following anti-PD-1/L1 failure in metastatic melanoma

This is a phase II clinical trial presenting the outcomes of the inaugural prospective trial evaluating the efficacy of combining ipilimumab at a low dose of 1 mg/kg with pembrolizumab in patients with advanced melanoma who experienced progression following treatment with anti-PD-1/L1 immunotherapy. The rationale behind this trial was to explore the therapeutic potential of this combination, particularly since the response to anti-CTLA-4 antibody alone post anti-PD-1/L1 antibody progression has historically been modest at 13%. Eligibility for the study was determined for patients who had advanced melanoma with progression on anti-PD-1/L1 antibody as their immediate preceding therapy, which could include combinations excluding anti-CTLA-4 antibodies. The treatment regimen consisted of intravenous pembrolizumab (200 mg) combined with ipilimumab (1 mg/kg) administered once every three weeks for a total of four doses, followed by maintenance pembrolizumab monotherapy, 200 mg once every 3 weeks for up to 2 years.

The primary objective of the trial, to determine the response rate (RR) as measured by irRECIST, was deemed achieved after initial results from 35 patients, prompting an expansion to a total of 70 patients to refine the estimate of response rate. Among these patients, 60 had previously been treated with an anti-PD-1 antibody alone, and 10 had received anti-PD-1/L1 antibody–based combinations. Additionally, 13 patients had shown progression in an adjuvant setting. The median duration of prior anti-PD-1/L1 antibody treatment was 4.8 months. The evaluation of responses revealed five complete responses and 15 partial responses, culminating in an irRECIST RR of 29% across the trial cohort. Furthermore, the median progression-free survival was recorded at 5.0 months, with a median overall survival of 24.7 months and a median response duration of 16.6 months. Notably, the analysis indicated no significant difference in the median duration of prior anti-PD1/L1 treatment until the initiation of the PD1 plus CTLA4 treatment between the responders and non-responders. Grade 3–4 drug-related adverse events were reported in 27% of the participants. Importantly, responses were observed across various tumor phenotypes, including PD-L1–negative, non-T-cell–inflamed, and intermediate tumors.

In conclusion, this pioneering study demonstrated that the combination of pembrolizumab and low-dose ipilimumab following the failure of anti-PD-1/L1 immunotherapy in melanoma showed significant anti-tumor activity and is tolerable. This suggests that the combination therapy could offer a viable treatment option for patients with advanced melanoma who do not respond to initial anti-PD-1/L1 therapies [29].

### Summary of treatment with nivolumab against metastatic melanoma

Another anti-PD-1 checkpoint inhibitor, nivolumab, has been used in the treatment of metastatic melanoma [30]. The region of the PD-L1, where pembrolizumab binds demonstrates a significantly higher congruence as compared to the binding region of nivolumab. Remarkably, the areas where pembrolizumab and nivolumab attach to the PD-1 molecule show virtually no overlap. Pembrolizumab, Keytruda®, is produced and distributed by Merck & Co., while Nivolumab, Opdivo®, is a product of Bristol Myers Squibb. Both medications share similarities in safety profiles and efficacy; however, they differ primarily in how often they are dosed, the medications they are combined with for treatment, and the specific cancers for which they have received approval. A retrospective comparison between pembrolizumab versus nivolumab against metastatic melanoma has shown no statistical difference in over-all survival, thus, supporting the current practice of choosing either of the two drugs based on the choice of the provider [31]. In this review [30], initial phase I/II studies have been discussed showing nivolumab to be both promising in terms of activity and safety [32], with its efficacy currently being further scrutinized in phase III trials against established therapies like chemotherapy and ipilimumab. Interestingly, combining nivolumab with ipilimumab appears to amplify its efficacy, albeit at the cost of increased toxicity. Unlike the transient benefits often seen with conventional cancer therapies in metastatic solid tumors, the therapeutic responses elicited by nivolumab tend to be long-lasting [30].

### Summary of clinical trial using nivolumab (against PD-1) and ipilimumab (against CTLA-4) versus nivolumab or ipilimumab alone against metastatic melanoma, CheckMate 067

Since nivolumab has been shown to be effective against metastatic melanoma [30–32], a randomized clinical trial, CheckMate 067, was conducted to evaluate the utility of combining nivolumab and ipilimumab. The 5-year outcomes of this study has been recently reported [33, 34]. In CheckMate 067, patients who had not previously received treatment for advanced melanoma, three groups were included: one received a combination of nivolumab (1 mg/kg) and ipilimumab (3 mg/kg) every three weeks for four doses, followed by nivolumab (3 mg/kg) every two weeks; another group was given nivolumab (3 mg/kg) every two weeks alongside a placebo; and the final group received ipilimumab (3 mg/kg) every three weeks for four doses plus a placebo. Treatment was carried out until the disease progressed, unacceptable side effects occurred, or consent was withdrawn. The two main outcomes included progression-free survival and overall survival, particularly comparing the nivolumab-plus-ipilimumab combination and nivolumab alone to ipilimumab alone.

The group receiving combination therapy with nivolumab and ipilimumab, the median overall survival exceeded 60 months, with the median not being reached even at a minimum follow-up of 60 months. In contrast, patients treated with nivolumab alone had a median overall survival of 36.9 months. The group treated with ipilimumab alone had the median overall survival at 19.9 months. The hazard ratio for death when comparing the combination therapy group (nivolumab plus ipilimumab) with the ipilimumab monotherapy group was 0.52. This indicated a 48% reduction in the risk of death with the combination therapy compared to ipilimumab alone. The hazard ratio for death when comparing the nivolumab monotherapy group with the ipilimumab monotherapy group was 0.63, suggesting a 37% reduction in the risk of death with nivolumab alone compared to ipilimumab alone. The 5-year overall survival rate was 52% for patients treated with the combination of nivolumab and ipilimumab. For those treated with nivolumab alone, the 5-year survival rate was 44%. The ipilimumab monotherapy group had a 5-year survival rate of 26%. The study reported no sustained deterioration in health-related quality of life during or after treatment with either the combination of nivolumab and ipilimumab or with nivolumab alone. Furthermore, no new late toxic effects were observed, indicating the long-term tolerability of these treatments.

These results suggest that combination therapy with nivolumab and ipilimumab significantly improves overall survival in patients, potentially those with advanced melanoma, compared to monotherapy with either drug. The considerable improvement in 5-year survival rates and the absence of new late toxic effects or sustained quality of life deterioration underscore the potential benefits and safety of combination therapy with these immunotherapeutic agents [34].

### Summary of first-line nivolumab plus ipilimumab with chemotherapy versus chemotherapy alone for metastatic non-small cell lung cancer in the CheckMate 9LA randomized trial

Combination of nivolumab and ipilimumab has been used in the CheckMate 9LA randomized clinical trial of metastatic non-small cell lung cancer. This study with a minimum follow-up of 47.9 months found that nivolumab plus ipilimumab combined with chemotherapy continued to significantly prolong overall survival as compared to chemotherapy alone in all randomized patients. The hazard ratio (HR) was 0.74 (95% CI: 0.63 to 0.87), with a 4-year OS rate of 21% versus 16%. This benefit was consistent regardless of tumor PD-L1 expression, with an HR of 0.66 (95% CI: 0.50 to 0.86) for patients with PD-L1 < 1% and an HR of 0.74 (95% CI: 0.60 to 0.92) for those with PD-L1 ≥ 1%. The efficacy also held across different histologies, with an HR of 0.64 (95% CI: 0.48 to 0.84) for squamous and 0.80 (95% CI: 0.66 to 0.97) for non-squamous. In patients who discontinued all components of nivolumab plus ipilimumab with chemotherapy due to treatment-related adverse events, the 4-year OS rate was 41%. After adjusting for the 36% of patients in the chemotherapy arm who received subsequent immunotherapy, the estimated HR for nivolumab plus ipilimumab with chemotherapy versus chemotherapy alone was 0.66 (95% CI: 0.55 to 0.80). No new safety signals were observed during the study. The authors concluded that this 4-year update demonstrateed that patients treated with nivolumab plus ipilimumab with chemotherapy continue to experience a long-term, durable efficacy benefit over chemotherapy alone, irrespective of tumor PD-L1 expression and histology. A greater relative overall benefit was observed after adjusting for the use of subsequent immunotherapy in the chemotherapy arm. These results further support the use of nivolumab plus ipilimumab with chemotherapy as a first-line treatment for patients with metastatic or recurrent non-small cell lung cancer, including those with PD-L1 < 1% or squamous histology, who represent populations with high unmet needs [35].

### LAG-3 as a new T cell checkpoint inhibition molecule

**Fig. 2:**
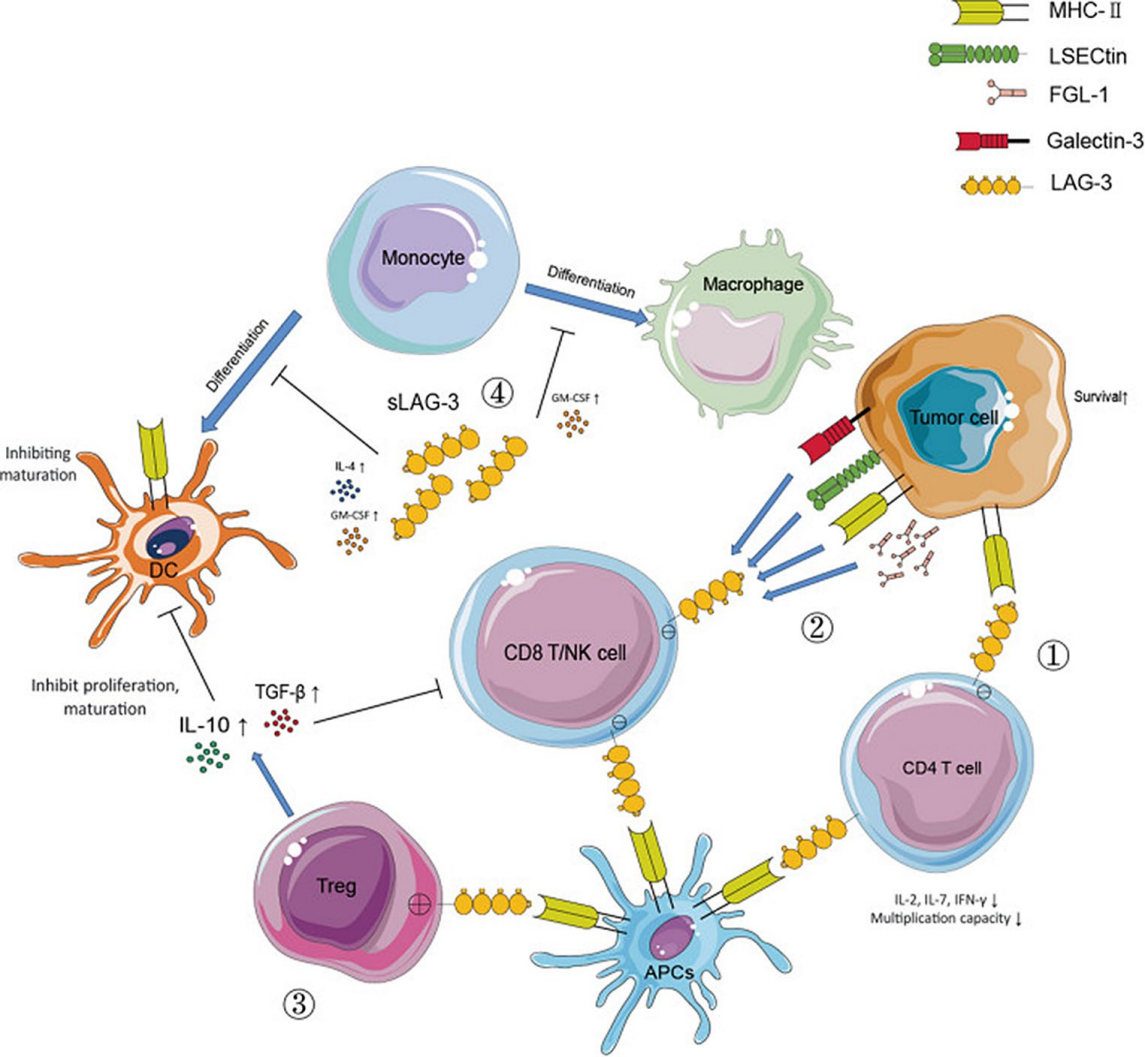
LAG-3 is a T-cell surface molecule which is closely related to CD4 on chromosome 12 with 20% homology in amino acid sequence by [36]. The mechanisms through which LAG-3 promotes immunosuppression within the tumor microenvironment (TME) are multifaceted: **1**) By engaging with MHC-II on CD4 T cells and tumor cells, LAG-3 curtails the proliferation and cytokine production of CD4 T cells, while the ensuing MHC-II signaling potentially aids tumor cell survival. **2**) The interaction between LAG-3 and molecules such as Galectin-3, LSECtin, and FGL-1, when it occurs between cytotoxic T lymphocytes (CTL)/natural killer (NK) cells and the TME, dampens the growth and lethal function of CTL/NK cells. **3**) When LAG-3 binds to MHC-II on regulatory T cells (Tregs) and either tumor cells or dendritic cells (DCs), it bolsters the stability and suppressive abilities of Tregs. Conversely, this interaction disrupts the maturation and antigen-presenting capabilities of DCs due to downstream MHC-II signaling. **4**) Soluble forms of LAG-3 (sLAG-3) present in the TME hinder the antigen presentation by monocyte-derived DCs (mDCs) and may also block the transformation of monocytes into DCs. This figure has been granted permission for reproduction in this review article [37]

LAG-3 is a T-cell surface molecule which may be potentially uses as a target for immunotherapy (Fig. 2). The combination of anti-LAG-3 (Relatlimab) with Nivolumab was tested in the RELATIVITY-047 trial, which demonstrated a significant improvement in progression-free survival (PFS) for patients with unresectable or metastatic melanoma when compared to Nivolumab alone [38]. This combination has been approved by the U.S. FDA for use in both adults and pediatric patients aged 12 years and above [39]. In the nivolumab + relatlimab group, grade 3/4 treatment-related adverse events were more frequent than the group receiving nivolumab. The findings from a recent updated report of the trial show the persistent efficacy of the nivolumab + relatlimab combination in providing a PFS benefit, although it did not meet the preplanned statistical threshold for overall survival (OS) [40].

The role of LAG-3 inhibitors, which function by binding to LAG-3 molecules or their ligands to prevent their interaction, is crucial in downregulating the inhibitory effect of LAG-3 on the immune system (Fig. 2). This mechanism is vital for enhancing the immune system’s ability to combat cancer [41]. The successful application of LAG-3 inhibitors like Relatlimab in combination with PD-1 inhibitors like Nivolumab opens up avenues for further exploration of other T-cell molecules that might play a role in immune response inhibition against cancer. The development of monoclonal antibodies targeting these molecules could potentially expand the arsenal of immunotherapeutic strategies against various cancers.

## The role of tumor infiltrating T cells (TILs) to fight against metastatic melanoma

TIL therapy is a form of adoptive immunotherapy that involves the infusion of activated T cells harvested from a patient’s own cancer, expanded in the lab, and then infused back into the patient with a therapeutic goal to kill the cancer. This adoptive immunotherapy has been developed and championed by Steve Rosenberg at the National Cancer Institute [42–50].

The process of TIL therapy starts with the resection of a patient’s autologous tumor, thus, the site of selecting the tumor is a surgical decision. The patient has unresectable cancer elsewhere, probably with multiple sites. The ideal site of tumor will be peripheral in location such as cutaneous or lymph node metastasis for melanoma in association with a deeper systemic site or sites, which are the targets for TIL adoptive immunotherapy. TILs represent a subset of immune cells that penetrate tumor tissue, playing a critical role in the body’s defense mechanism against cancer. These cells, which predominantly include various T cell subsets, are instrumental in targeting and eliminating cancer cells through several key actions. It is assumed that TILs possess the unique ability to recognize antigens present on the surface of cancer cells. These antigens may be proteins that are either abnormally overexpressed by cancer cells or uniquely characteristic of the cancer cells. The T cells within the TIL population can specifically latch onto these antigens, displayed by the tumor cells’ major histocompatibility complex (MHC), triggering the T cells’ activation. Once activated, a specific group of TILs, known as cytotoxic T lymphocytes (CTLs), can directly attack and kill the cancer cells. They achieve this by secreting substances like perforin and granzymes that prompt the cancer cells to undergo apoptosis, or cell death. Additionally, they can initiate the death of cancer cells by activating certain pathways through binding to death receptors with their ligands, such as Fas ligand (FasL). Beyond their direct attack on cancer cells, TILs also secrete cytokines, powerful signaling molecules that amplify the immune response against the cancer. Interferon-gamma (IFN-γ), one of the cytokines produced, directly inhibits cancer growth, enhances the visibility of cancer cells to the immune system by upregulating MHC molecule expression, and activates other immune cells like macrophages and NK cells to join the fight against the cancer.

TILs further orchestrate an extensive immune response by activating other immune cells within the cancer microenvironment. Helper T cells within the TIL population assist in activating both CTLs and B cells, the latter of which can produce antibodies targeting cancer antigens, facilitating the destruction of cancer cells by other immune mechanisms such as antibody-dependent cellular cytotoxicity (ADCC). Moreover, TILs are adept at counteracting the immunosuppressive environments created by cancer cells to evade immune detection. Through their multifaceted activities, TILs can breach these defenses, enabling an effective immune assault on the cancer cells. The presence of TILs within the cancer microenvironment has been correlated with improved prognoses in various cancers, highlighting their ability to mitigate cancer-induced immune suppression. TILs also exhibit adaptability, with the capacity to evolve into memory cells after their initial encounter with cancer antigens. These memory cells are primed to respond rapidly to future instances of the same cancer antigens, providing a long-lasting surveillance mechanism against cancer recurrence.

Therefore, the presence and activities of TILs within the cancer cells are essential for mounting an effective immune response against cancer. Understanding the biology of TILs has led to the development of innovative cancer treatments that aim to boost the function and presence of TILs, such as immune checkpoint inhibitors and adoptive cell transfer therapies, marking a promising direction in cancer therapy.

It is important to expand the TILs ex vivo outside of the inhibitory cancer microenvironment with, perhaps, interleukin 2 (IL-2), to expand TILs to be infused back to the patient, so that these activated TILs in sufficient number will be directed against the metastatic sites, hopefully to destroy the cancer. Based on mouse data, Rosenberg has introduced a lymphodepleting regimen consisting of cyclophosphamide [51], prior to TIL infusion, to overcome the immunosuppression condition of the patient. In addition, high-dose IL-2 is given along with the TIL infusion. An excellent review of this subject has been summarized by Yang and Rosenberg [52]. Surgical consideration for tumor tissue for the preparation of TIL infusion was presented at the 9th International Cancer Metastasis Congress in San Francisco in May 2023 by John Mullinax [53]. In the current landscape of treating metastatic melanoma patients who have failed checkpoint inhibitors and BRAF +/- MEK targeted therapy, objective responses were observed using TIL infusion by Sarnaik et al. [54]. A phase II open-label, single-arm and multicenter study was conducted in advanced melanoma patients who had been previously treated with checkpoint inhibitor(s) and BRAF with or without MEK targeted agents. Lifileucel is a preparation utilizing TILs derived from the patient’s own body. It was manufactured from harvested melanoma specimens in central Good Practice facilities using a streamlined 22-day process. A single infusion of lifileucel (1 × 109– 150 × 109 cells) was administered after approximately 24 h from the last dose of fludarabine (immunodepletion agent). A short course of bolus IL-2 (600,000 IU/kg) was infused every 8–12 h for up to six doses, starting within 3–24 h of completing lifileucel infusion. The study’s main goal was to measure the objective response rate as determined by the investigators according to the Response Evaluation Criteria in Solid Tumors (RECIST), version 1.1, in 66 patients being included in the study. The objective response rate was found to be 36% (with a 95% confidence interval [CI] of 25 to 49), including two complete responses and 22 partial responses. The disease control rate stood at 80% (95% CI, 69 to 89). The median duration of response had not been reached by the end of the 18.7-month median follow-up period (which ranged from 0.2 to 34.1 months). Specifically, in the subgroup of patients who were primarily refractory to anti-PD-1 or PD-L1 therapy, the objective response rate and disease control rate were 41% (95% CI, 26 to 57) and 81% (95% CI, 66 to 91), respectively. The safety profile was in line with the expected adverse events associated with nonmyeloablative lymphodepletion and interleukin-2 treatment.

In conclusion, the treatment known as Lifileucel showed durable responses and addressed a significant unmet need for patients with metastatic melanoma who have exhausted their treatment options after standard therapy, including those who did not respond to initial anti-PD-1 or PD-L1 therapy [54].

TILs have shown promising results in heavily treated patients including metastatic melanoma [46, 54–59], non-small cell lung cancer [60, 61], cervical cancer [58, 62] and head and neck cancer [58, 63]. In some patients, TIL therapy has led to significant tumor shrinkage or even complete remission. However, it’s important to note that this treatment approach is still considered experimental and is not widely available. It is typically reserved for patients who have not responded to other standard treatments or have no viable alternative options. The critical part of TIL adoptive therapy rests on the ability of expanding the T cells ex vivo. Numerous laboratory techniques and reagents have been introduced to enhance and improve the expansion of TILs for so that TILs can be more reliably expanded for infusion therapy [53]. Recent improved culture techniques have resulted in over 95% success rate of generating TILs for infusion [64]. FDA has recently approved the lifileucel treatment (Amtagvi, Iovance Biotherapeutics, Inc.) for unresectable metastatic melanoma (https://www.fda.gov/drugs/resources-information-approved-drugs/fda-grants-accelerated-approval-lifileucel-unresectable-or-metastatic-melanoma#:~:text=On%20February%2016%2C%202024%2C%20the%20Food%20and%20Drug,BRAF%20inhibitor%20with%20or%20without%20a%20MEK%20inhibitor) with failure to previous treatment with a PD-1 blocking antibody and/or a BRAF inhibitor with or without a MEK inhibitor (if BRAF is V600 positive).

In addition to infusion of TILs as a form of adoptive immunotherapy, other approaches may include gene-modified T-cell receptor therapy and chimeric antigen receptor-modified T cells [65–67]. These approaches are not discussed in this review article.

## Comparison between neoadjuvant and adjuvant therapy for resectable metastatic cancer

Neoadjuvant drug therapy is administered prior to surgery with the aim of reducing the tumor size or halting the cancer’s progression, thereby facilitating a less complex and more successful surgical procedure. Conversely, adjuvant therapy is given post-surgery to eliminate any residual cancer cells, aiming to lower the risk of the cancer recurrence. In this study pembrolizumab was used to compare its effectiveness in neoadjuvant versus adjuvant setting in advanced melanoma. A phase 2 trial (S1801) was conducted. Melanoma patients with clinically detectable stage IIIB to IVC being amenable to surgical resection were randomized to three doses of neoadjuvant pembrolizumab, surgery, and 15 doses of adjuvant pembrolizumab (neoadjuvant–adjuvant group) or to surgical resection followed by pembrolizumab (200 mg intravenously every 3 weeks for a total of 18 doses) for approximately 1 year or until disease recurred or the development of unacceptable toxic effects developed (adjuvant-only group). The study involved 154 patients in the neoadjuvant-adjuvant group and 159 patients in the adjuvant-only group, with a median follow-up duration of 14.7 months. The neoadjuvant-adjuvant group showed significantly longer event-free survival compared to the adjuvant-only group, with statistical significance achieved (*P* = 0.004 by the log-rank test). This indicated that patients who received pembrolizumab both before and after surgery had a lower rate of cancer recurrence or progression within the study period. At 2 years, the event-free survival rate was 72% (with a 95% confidence interval [CI] of 64–80%) in the neoadjuvant-adjuvant group, compared to 49% (95% CI, 41–59%) in the adjuvant-only group. This study showed a substantial improvement in outcomes for patients receiving the combination approach. The incidence of treatment-related adverse events of grades 3 or higher (which are more severe side effects) was relatively low and comparable between the two groups, with 12% in the neoadjuvant-adjuvant group and 14% in the adjuvant-only group suggesting that the addition of neoadjuvant pembrolizumab does not significantly increase the risk of severe adverse events. The study concluded that for patients with resectable stage III or IV melanoma, undergoing pembrolizumab treatment both before and after surgery significantly prolonged event-free survival compared to receiving pembrolizumab only after surgery. Additionally, the study did not identify any new toxic effects associated with this treatment strategy. These findings are significant as they suggest that a neoadjuvant-adjuvant treatment approach with pembrolizumab can offer a more effective strategy for improving outcomes in patients with advanced melanoma, without introducing additional severe side effects. This may potentially represent a shift in the treatment paradigm for this patient population [68].

Similarly, in the CheckMate 816 open-label phase 3 clinical trial for resectable stage IB to IIIA non-small cell lung cancer, neoadjuvant nivolumab plus chemotherapy showed better event-free survival than adjuvant chemotherapy. The patients were randomly assigned to receive either nivolumab plus platinum-based chemotherapy or platinum-based chemotherapy alone before undergoing surgical resection. Event-free survival and pathological complete response (defined as 0% viable tumor cells in resected lung and lymph nodes), both were assessed by a blinded independent review. Median event-free survival was significantly longer in the nivolumab plus chemotherapy group (31.6 months) compared to the chemotherapy-alone group (20.8 months), with a hazard ratio of 0.63, indicating a 37% reduction in the risk of disease progression, recurrence, or death. A significantly higher percentage of patients achieved a pathological complete response in the nivolumab plus chemotherapy group (24.0%) compared to the chemotherapy-alone group (2.2%). At the first interim analysis, the hazard ratio for death was 0.57, suggesting a trend towards improved survival with the addition of nivolumab, although this did not meet the predefined criteria for statistical significance.

Most patients in both groups underwent surgery, with 83.2% in the nivolumab-plus-chemotherapy group and 75.4% in the chemotherapy-alone group proceeding to resection. The incidence of grade 3 or 4 treatment-related adverse events was similar between the two groups, with 33.5% in the nivolumab-plus-chemotherapy group and 36.9% in the chemotherapy-alone group. The authors have concluded that neoadjuvant treatment with nivolumab plus chemotherapy significantly improves event-free survival and increases the rate of pathological complete response in patients with resectable NSCLC compared to chemotherapy alone. The addition of nivolumab to chemotherapy does not lead to an increase in serious adverse events and does not impede the ability to perform surgery. This study supports the use of nivolumab in combination with platinum-based chemotherapy as a neoadjuvant treatment for early-stage NSCLC, offering patients a better chance of extended event-free survival and a higher likelihood of achieving a complete pathological response, without compromising safety or the feasibility of subsequent surgical intervention [69]. 

### Summary of the study on pembrolizumab versus placebo as adjuvant therapy in resected stage IIB or IIC melanoma: phase III KEYNOTE-716 trial

Luke et al. [70] presented the final analysis of distant metastasis-free survival (DMFS) for the KEYNOTE-716 study, which involved 976 melanoma patients with stage IIB and IIC with a negative sentinel lymph node biopsy being randomly assigned to either pembrolizumab (487 patients) or placebo (489 patients). As of January 4, 2023, after a median follow-up of 39.4 months, neither group reached the median DMFS. The estimated 36-month DMFS was 84.4% for pembrolizumab and 74.7% for placebo, showing a significant benefit for pembrolizumab (hazard ratio [HR], 0.59). Similarly, the estimated 36-month recurrence-free survival (RFS) was 76.2% for pembrolizumab and 63.4% for placebo (HR, 0.62). These results were consistent across most subgroups, including patients with stage IIB and IIC melanoma. For patients with stage IIB melanoma, neither the pembrolizumab nor placebo groups reached a median distant metastasis-free survival (DMFS). At 36 months, the DMFS rate was 86.7% for pembrolizumab compared to 78.9% for placebo, showing a significant benefit with pembrolizumab (hazard ratio [HR], 0.62). In stage IIC melanoma patients, the median DMFS was also not reached in both groups, with a 36-month DMFS rate of 80.9% for pembrolizumab and 68.1% for placebo, again favoring pembrolizumab (HR, 0.57).

Pembrolizumab’s safety profile was manageable and in line with prior studies. These findings support the continued use of pembrolizumab as adjuvant therapy for patients with resected stage IIB or IIC melanoma.

In this randomized, double-blind, phase III KEYNOTE-716 trial for resected stage IIB or IIC melanoma, pembrolizumab as adjuvant therapy significantly improved RFS in the first interim analysis and DMFS in the third interim analysis as compared to placebo. These results led to the approval of pembrolizumab as adjuvant therapy for this condition by various regulatory bodies, including the US FDA and European Medicines Agency. This report includes the fourth interim analysis of KEYNOTE-716, with final DMFS and updated RFS outcomes [70].

## Failure rate of checkpoint inhibition immunotherapy: possible mechanisms of failure

In most solid tumors, response rates to checkpoint inhibition immunotherapy vary between 15% and 30% [71], while in melanoma [34] and microsatellite instability-high (MSI-H) colon cancer [72], the response rates are notably higher, ranging from 45 to 60%. Despite the impressive successes of several recent immunotherapy trials, many cancer patients either do not respond to these treatments, or they experience only temporary benefits before the disease recurs. This recurrence is primarily due to rapidly developing resistance to the therapies. The nature of this resistance, whether primary or acquired, remains largely unexplored and poorly understood, making it a significant barrier to effective treatment. Further detailed research is essential to address this issue, particularly at the molecular level, where genetic and protein-related mechanisms are likely to play key roles. From what we understand of the complex nature of the immunological response, the resistance mechanisms to immune checkpoint inhibitors are expected to be even more complex and multifaceted. These mechanisms likely involve a variety of factors within the cancer microenvironment including gene expression, cellular metabolism, and the presence or absence of inflammation, as well as angiogenesis and tumor neovascularization [73]. Additional contributors include factors from host cells, as well as broader influences like older age, biological sex, dietary habits, various hormones, pre-existing health conditions, and the composition of the gut microbiome. Each of these factors can play a significant role in determining how effectively the immune system can respond to challenges such as cancer [74]. Among the recently identified co-inhibitory receptors such as Lag-3, Tim-3 and TIGIT [37, 75], Lag-3 is the only receptor that has been found to enhance the activity of nivolumab as noted in the RELATIVITY-047 trial as mentioned above [38]. However, as a monotherapy, Lag-3 is not as effective in comparison to CTLA-4 or PD-1/PD-L1.

## The role of mRNA vaccine in the treatment of melanoma patients following resection of their metastatic melanoma

Checkpoint inhibition immunotherapy is now a standard preventive treatment option for resectable stage IIB–IV melanoma, but the risk of cancer returning remains high. The KEYNOTE-942 phase 2b, open-label, randomized trial aimed to investigate the effectiveness of mRNA-4157 (V940), a new personalized cancer vaccine using mRNA technology, in combination with pembrolizumab versus pembrolizumab alone in patients from the USA and Australia who had undergone surgery for high-risk melanoma. Patients with melanoma stages IIIB–IV who had undergone surgery were randomly assigned in a 2:1 ratio to receive the combination therapy or pembrolizumab alone. The mRNA-4157 vaccine was given as an injection into the muscle (up to nine doses), and pembrolizumab was given intravenouly (up to 18 doses), both in 3-week cycles. The main goal was to measure the time until the cancer returned in all patients who were treated. This trial is still ongoing and is registered under NCT03897881 at ClinicalTrials.gov. Between July 18, 2019, and September 30, 2021, 157 patients were enrolled, with 107 receiving the combination therapy and 50 receiving pembrolizumab alone. The median time the patients were followed up was 23 months for the combination group and 24 months for the monotherapy group. The combination therapy showed a trend towards longer time without cancer recurrence compared to monotherapy (with a hazard ratio for recurrence or death being 0.561 [95% CI 0.309–1.017]; *p* = 0.053), and fewer patients experienced cancer recurrence or death (22% in the combination group vs. 40% in the monotherapy group). The 18-month rate of not having cancer recurrence was 79% for the combination group compared to 62% for the monotherapy group. Most side effects from treatment were mild to moderate. Severe treatment-related side effects occurred in 25% of patients in the combination group and 18% in the monotherapy group, with no severe side effects related to mRNA-4157. The frequency of side effects related to the immune system was similar in both groups (36%). The combination of the mRNA-4157 vaccine and pembrolizumab extended the time without cancer recurrence compared to pembrolizumab alone in patients with surgically removed high-risk melanoma and had a tolerable safety profile. These findings suggest that a personalized mRNA-based cancer vaccine could be advantageous as an additional preventive treatment [76].

The above immunotherapeutic clinical trials are summarized in Table 1 to show the remarkable results against metastatic cancer using immune checkpoint inhibitors alone or with other agents since 2010.

**Table 1:** Summary of the remarkable results of checkpoint inhibitors alone or with other agents against metastatic cancer or in an adjuvant setting since 2015

Clinical Trials	Summary	Reference
Clinical trials using ipilimumab against metastatic melanoma based on checkpoint inhibition of CTLA-4	A total of 4,846 patients with advanced melanoma were treated with ipilimumab and reached with a plateau at 21% in the overall survival curve beginning around year 3. These data add to the evidence supporting the durability of long-term survival in ipilimumab-treated patients with advanced melanoma.	[25]
Comparison of Ipilimumab alone, Ipilimumab plus gp 100 peptide vaccine and gp 100 peptide vaccine alone in the treatment of metastatic melanoma	Ipilimumab, either alone or combined with a gp100 peptide vaccine, has shown to enhance overall survival in patients with previously treated metastatic melanoma, compared to gp100 alone. Although adverse effects from the treatment can be severe and long-lasting, they are mostly reversible with proper treatment. Thus, gp100 peptide vaccine has shown no effect on survival.	[26]
Summary of clinical trials using pembrolizumab against metastatic melanoma based on checkpoint inhibition of PD-1	In this study, 173 patients received pembrolizumab 2 mg/kg (*n* = 89) versus 10 mg/kg (*n* = 84) with a median follow-up of 8 months. The overall response rate was 26% at both doses (difference 0%, 95% CI − 14 to 13; *p* = 0.96). Safety profiles in the 2 mg/kg and 10 mg/kg groups were similar and no drug-related deaths with the treatment being well tolerated. The authors indicated their results support the conclusion that pembrolizumab at 2 mg/kg or 10 mg/kg every 3 weeks might be effective in patients who had failed ipilimumab treatment.	[27]
Clinical trial comparing pembrolizumab (against PD-1) and ipilimumab (against CTLA-4) against metastatic melanoma	This study underscores pembrolizumab’s superiority over ipilimumab in enhancing progression-free survival, overall survival, and response rates among patients with advanced melanoma. Pembrolizumab also exhibited a more favorable safety profile, characterized by a lower incidence of serious adverse effects. Notably, the efficacy of pembrolizumab remained consistent across its different dosing schedules, suggesting flexibility in treatment management.	[28]
Summary of clinical trial using pembrolizumab (against PD-1) and ipilimumab (against CTLA-4) against metastatic melanoma following anti-PD-1/L1 failure in metastatic melanoma	This study demonstrated that the combination of pembrolizumab and low-dose ipilimumab following the failure of anti-PD-1/L1 immunotherapy in melanoma shows significant anti-tumor activity and is tolerable. This suggests that the combination therapy could offer a viable treatment option for patients with advanced melanoma who do not respond to initial anti-PD-1/L1 therapies.	[29]
Treatment with nivolumab against metastatic melanoma	Nivolumab, a monoclonal antibody targeting the PD-1 receptor, enhances antitumor immunity by inhibiting this crucial suppressor of T-cell activation. This review summarizes phase I/II studies on Nivolumab showing efficacy against metastatic melanoma as well as phase III trials currently comparing nivolumab to standard treatments like chemotherapy and ipilimumab. Combining nivolumab with ipilimumab may boost its efficacy, although it also increases toxicity. Responses to nivolumab tend to be durable.	[30]
Clinical trial using nivolumab (against PD-1) and ipilimumab (against CTLA-4) versus nivolumab or ipilimumab alone against metastatic melanoma, CheckMate 067	This study showed that combination therapy with nivolumab and ipilimumab significantly improves overall survival in patients, potentially those with advanced melanoma, compared to monotherapy with either drug. The considerable improvement in 5-year survival rates and the absence of new late toxic effects or sustained quality of life deterioration underscore the potential benefits and safety of combination therapy with these immunotherapeutic agents.	[34]
First-line nivolumab plus ipilimumab with chemotherapy versus chemotherapy alone for metastatic non-small cell lung cancer in the CheckMate 9LA randomized trial	The authors concluded that this 4-year update demonstrated that patients treated with nivolumab plus ipilimumab with chemotherapy continue to experience a long-term, durable efficacy benefit over chemotherapy alone, irrespective of tumor PD-L1 expression and histology. A greater relative overall benefit was observed after adjusting for the use of subsequent immunotherapy in the chemotherapy arm.	[35]
LAG-3 as a new T cell checkpoint inhibition molecule	The role of LAG-3 inhibitors, which function by binding to LAG-3 molecules or their ligands to prevent their interaction, is crucial in downregulating the inhibitory effect of LAG-3 on the immune system. The successful application of LAG-3 inhibitors like Relatlimab in combination with PD-1 inhibitors like Nivolumab opens avenues for further exploration of other T-cell molecules that might play a role in immune response inhibition against cancer. A recent updated report of the trial show the persistent efficacy of the nivolumab + relatlimab combination in providing a PFS benefit, although it did not meet the preplanned statistical threshold for overall survival.	[38]
The role of tumor infiltrating T lymphocytes (TILs) to fight against metastatic melanoma	TIL (tumor-infiltrating lymphocyte) therapy is a type of adoptive immunotherapy pioneered by Steve Rosenberg at the National Cancer Institute in the late 1980s. It involves extracting T cells from a patient’s tumor, expanding them in a laboratory, and then reinfusing them into the patient. The goal is for these activated T cells to target and destroy cancer cells. This approach leverages the patient’s own immune system for therapeutic purposes. In a recent TIL adoptive cell therapy for melanoma patients who have failed checkpoint inhibition. The objective response rate was 41% and disease control rate was 81% FDA has just approved the TIL therapy on 2/19/2024.	[42, 54]
Clinical trial on pembrolizumab versus placebo as adjuvant therapy in resected Stage IIB or IIC melanoma: phase III KEYNOTE-716 trial	In this randomized, double-blind, phase III KEYNOTE-716 trial for resected stage IIB or IIC melanoma, pembrolizumab as adjuvant therapy significantly improved RFS in the first interim analysis and DMFS in the third interim analysis compared to placebo. These results led to the approval of pembrolizumab as adjuvant therapy for this condition by various regulatory bodies, including the US FDA and European Medicines Agency. This report includes the fourth interim analysis of KEYNOTE-716, with improved DMFS and RFS outcomes.	[70]
The role of mRNA vaccine in the treatment of melanoma patients following resection of their metastatic melanoma (Keynote-942 study)	The KEYNOTE-942 phase 2b, open-label, randomized trial aimed to investigate the effectiveness of mRNA-4157 (V940), a new personalized cancer vaccine using mRNA technology, in combination with pembrolizumab versus pembrolizumab alone in patients from the USA and Australia who had undergone surgery for high-risk melanoma. The combination of the mRNA-4157 vaccine and pembrolizumab extended the time without cancer recurrence compared to pembrolizumab alone in patients with surgically removed high-risk melanoma and had a tolerable safety profile.	[76]
Comparison between neoadjuvant and adjuvant therapy for resectable metastatic cancer	These findings are significant as they suggested that a neoadjuvant-adjuvant treatment approach with pembrolizumab could offer a more effective strategy for improving outcomes in patients with advanced melanoma, without introducing additional severe side effects. This could potentially represent a shift in the treatment paradigm for this patient population. Similarly, in the CheckMate 816 clinical trial for resectable non-small cell lung cancer, neoadjuvant nivolumab plus chemotherapy showed better event-free survival than adjuvant chemotherapy.	[68, 69]

## Diversity of T cell receptor repertoire against cancer

This portion of the review has been presented by Dr. Mark Davis at the 9th International Cancer Metastasis Congress in San Francisco in May 2023. The T cell receptor (TCR) diversity is vast with − 10^15^ possible combinations of alpha and beta chains. The diversity of TCR is fundamental to the adaptive immune system’s capability to detect and combat a myriad array of pathogens by enabling each T cell to uniquely recognize specific antigens. This remarkable diversity stems from the variable-diversisty-joining rearrangement or V(D)J recombination mechanism, which results in the highly diverse repertoire of immunoglobulins and TCRs. T cell receptor (TCR) genes are akin to immunoglobulin genes because they also have multiple V (variable), D (diversity), and J (joining) gene segments in their beta chains, and V and J segments in their alpha chains. These segments undergo rearrangement during lymphocyte development, equipping each cell with a unique antigen receptor. In this regard, the T cell receptor is structurally comparable to the antigen-binding fragment of an antibody, with both belonging to the immunoglobulin superfamily. 

The genesis of TCR diversity primarily occurs through V(D)J recombination during T cell development in the thymus. This process starts when Recombination Activating Gene (RAG) proteins create double strand breaks near the V, D, and J segments. Such breaks facilitate the diverse combination of these gene segments by joining them together, with the unwanted DNA being either removed or inverted. For the β, γ, and δ chains, this recombination includes both D to J and V to DJ joining, whereas for the α chain, it involves V to J joining. The diversity of TCRs is further enhanced at the points where these segments join, through either the addition or deletion of nucleotides. This is done by terminal deoxynucleotidyl transferase, which inserts N-nucleotides at the junctions, vastly increasing the variability in the antigen-binding portion of the TCR.

An additional layer of TCR variability, known as combinatorial diversity, comes from the random assembly and pairing of various α and β chains (or γ and δ chains) from the repertoire created by V(D)J recombination, generating a multitude of possible TCR combinations within the T cell population. To ensure that each T cell exhibits a TCR with a singular specificity, a mechanism called allelic exclusion operates to permit only one allele of the TCR α and β chain genes to undergo productive rearrangement in each cell. This is vital for preventing the emergence of TCRs with mixed specificities, which could recognize self-antigens, leading to autoimmunity. The extensive TCR repertoire produced through these processes is then refined within the thymus via selection mechanisms. Through positive selection, T cells whose TCRs recognize self-MHC molecules presenting foreign peptides are preserved, while those binding too strongly to self-peptides presented by self-MHC are deleted via negative selection. This ensures that selected TCR repertoire is capable of foreign antigen recognition while being self-tolerant. In essence, the adaptive immune system’s ability to identify and neutralize a broad spectrum of pathogens while avoiding self-reactivity is achieved through a complex interplay of V(D)J recombination, enhanced junctional and combinatorial diversities, and meticulous thymic selection, culminating in a highly diverse and specific T cell repertoire [77–80]. Thus, unrelated individuals and identical twins have almost entirely non-identical repertoires, even if they are making T cell responses against the same antigens bound to the same MHC molecules. Sequence data from the human genome project may generate millions and millions of TCR sequences from blood, lymph node and tumor samples. The challenge is identifying the relevant TCRs that recognise antigens such as those from cancer or infectious agents.

Glanville, Davis and colleagues [81] have pioneered a method to pinpoint the basic elements necessary for the specificity of T-cell receptors (TCRs) for antigens. This specificity is achieved through careful analysis of TCR sequences using a panel of peptide and major histocompatibility complex (pMHC)-tetramer-sorted cells and available TCR-peptide MHC structural data. This work led to the creation of the Grouping of Lymphocyte Interactions by Paratope Hotspots (GLIPH) algorithm. GLIPH classifies TCRs based on their likely shared specificities, which are identified through recurring motifs and overall similarity in complementarity-determining region 3 (CDR3) sequences. The authors effectively demonstrated GLIPH’s ability to classify TCRs from different individuals with a common specificity. They have found that repeated CDR3 motifs are critical for the formation of these TCR clusters, often serving as interaction points with antigenic peptides. To confirm the effectiveness of GLIPH, they examined 5,711 TCRβ chain sequences from CD4 T cells from 22 individuals with dormant M. tuberculosis infection, identifying 141 distinct groups of TCR specificities. Sixteen groups consisting of TCRs from different individuals were identified. These groups often share HLA alleles that help predict HLA restrictions. A broad spectrum of M. tuberculosis T cell epitopes are identified, which are elements of MHC-bound peptide sequences which are recognized by their respective TCRs. Additional experiments involving mutagenesis and the creation of new TCRs confirmed the importance and sufficiency of the motifs recognized by GLIPH for recognition of shared antigens. GLIPH achieves several important goals: (1) It establishes a method for grouping TCR sequences that are likely to recognize the same peptide-MHC combination; (2) It extracts motifs associated with specific antigen interactions, helping to discern vaccination and infection history; (3) It identifies common popular themes among different individuals; (4) It assesses the diversity of T cell responses; and (5) It facilitates analysis of α-β chain T cell responses based on sequence data, independent of antigen and MHC knowledge. Using GLIPH, the researchers identified five TCR beta-specific groups from approximately 5,700 sequences from M. tuberculosis-specific CD4 + cells, most of which had the same peptide-MHC specificity [81]. Huang and Davis et al. subsequently improved the program to be able to analyze more than 10 million TCR sequences [82]. In essence, GLIPH represents a breakthrough in studying T cell responses, accelerating the identification of cognate ligands and enriching our understanding of T cell specificity dynamics.

Simoni and colleagues [83] explored the various successes of immunotherapies such as checkpoint inhibitor therapy in reactivating T cell-driven anti-cancer immune responses. However, successful responses occur only in some patients and types of cancer [42, 84–86]. They note that the unpredictable effectiveness of these therapies in different patients and cancer types may stem from diversity in immune cell composition and phenotypic variation in tumor-infiltrating lymphocytes (TILs). The unpredictable responses to checkpoint inhibition immunotherapy may partly be due to tumor heterogeneity as well as to heterogeneous immune responses relating to TILs within individual patient and among patients [87, 88]. Although it is recognized that T cells directed against neoantigens derived from tumor mutations are critical for fighting tumors [84, 86, 89–92] the exact antigens targeted by the various TILs are largely unknown. The authors found that in human lung and colorectal cancers, CD8 + TILs target not only tumor antigens, including neoantigens, but also multiple non-cancer-related epitopes, such as Epstein-Barr virus, human cytomegalovirus, or influenza virus epitopes. These “bystander” CD8 + TILs, although phenotypically diverse and similar to tumor-specific cells, are characterized by a lack of CD39 expression.

CD39, also known as Cluster of Differentiation 39, is an enzyme that plays a pivotal role in controlling purinergic signaling within immune cells. Together with CD73, CD39 acts to transform adenosine triphosphate (ATP) first into adenosine diphosphate (ADP) and then into cyclic adenosine monophosphate (AMP). This sequence of reactions ultimately results in the production of an immunosuppressive variant of adenosine. Through this mechanism, CD39 and CD73 function as “immunological switches,” transitioning the immune response from a proinflammatory state to an anti-inflammatory one. In cases of colorectal and lung cancer, the lack of CD39 in CD8 + TILs means that tumor sites show no signs of sustained antigen exposure, suggesting their role as irrelevant bystanders. Furthermore, significant differences in CD39 expression were observed across patients, with some patients exhibiting a high proportion of CD39-CD8 + TILs. The amount of CD39 expression in these TILs correlates with important clinical factors, such as the mutational status of the epidermal growth factor receptor in lung cancer. The study by Simoni et al. highlights that not all T cells within a cancer population are suitable for fighting cancer antigens. This study demonstrates that assessing CD39 performance can effectively quantify or isolate these bystander T cells. This approach provides new ways to understand the complex dynamics within the cancer microenvironment and its impact on the efficacy of cancer immunotherapy [83].

Using non-small cell lung cancer (NSCLC) as a model, Chiou, Tseng and Davis et al. conducted an extensive analysis to pinpoint the role of disease-associated TCRs with common antigen specificity in the lung cancer patients [93]. They examined a large set of 778,938 TCRβ chain sequences from 178 NSCLC patients using the advanced GLIPH2 (Group of Lymphocyte Interactions with Paratope Hotspots 2) algorithm. This in-depth analysis identified more than 66,000 shared specificity groups, 435 of which showed clonal expansion and were more prevalent in lung cancer cells than in adjacent lung tissue. To identify the epitopes of these tumor-enriched TCR groups, they utilized a yeast peptide-HLA A*02 display library. This showed that one of the groups identified a peptide from TMEM161A, an epithelial protein commonly overexpressed in tumors. Interestingly, this group also reacted to cross-reactive epitopes from Epstein-Barr virus and E. coli, suggesting a potential cross-reactivity phenomenon. These findings suggest that the presence of virus-specific T cells in infiltrates may be due in part to this cross-reactivity, suggesting that this mechanism may be common in various cancers. The methods and analytical tools developed in this study and the specificity populations identified provide a valuable resource for understanding the dynamics of T cell responses in cancer. This study opens new avenues for exploring how TCR specificity and cross-reactivity influence immune responses in the cancer microenvironment [93].

In a melanoma model, Huuhtanen, Davis and their colleagues [94] conducted an in-depth examination of antigen-specific T cell responses, focusing on melanoma cases. The study involved analysis of three T-cell receptor (TCR) repertoire data types: antigen-specific TCRs, overall TCR repertoires, and single-cell RNA sequencing combined with TCRαβ sequencing. The analysis included 515 patients with primary or metastatic melanoma and compared them with 783 patients without the disease. A striking finding of the study is that although TCRs directed against melanoma-associated antigens (MAAs) have individual specificities, they exhibit some sequence similarity. These similarities were used to create predictive models that identify anti-MAA T cells. The prevalence of these anti-MAA T cells distinguishes melanoma patients from unaffected individuals and predicts the likelihood of metastatic recurrence in primary melanoma cases. Furthermore, this study shows that anti-MAA T cells have stem cell-like properties and frequently interact with regulatory T cells and tumor cells [94]. These interactions occur primarily through the Galectin9-TIM3 [95] and PVR-TIGIT [96] pathways. Importantly, in patients who showed a positive response to treatment, there was a significant increase in the number of proliferating anti-MAA clones following anti-PD1 (and anti-CTLA4) treatment, as well as a reversal of the exhausted state of these clones of T cells. This systemic immunology approach by Huuhtanen, Davis, and colleagues [94] represents an important step forward in understanding antigen-specific responses in human disease, particularly cancer immunotherapy. Their findings have the potential to advance personalized medicine approaches and develop more targeted therapies for melanoma and possibly other diseases [94].

## Conclusion and future perspectives

This review article summarizes the discovery of T cells and recent developments of immunotherapy against cancer based on the molecular mechanisms of checkpoint inhibition. Humans can produce a variety of TCRs, each targeting a unique antigen. This broad array is critical to the immune system’s ability to detect and fight a variety of pathogens, including abnormal cells that cause cancer. A broader TCR repertoire increases the likelihood that T cells will recognize and attack cancer cells more effectively. To date, the cancer neoantigens are still poorly defined, and yet, it is assumed that they are the targets for the immune system. The recent clarification of the molecular mechanisms governing the immune responses to cancer by Allison and Honjo [97], coupled with the deployment of immune checkpoint inhibitors against cancer [98], have solidified the critical role of the immune system in the regulation of cancer. The fact that by inhibiting the checkpoint mechanisms resulting in destruction of cancer cells by activated T cells as shown in successful clinical trials being summarized in this review article securely establishes the importance of the immune system to fight against cancer. The definition of cancer neoantigens and the T cell receptor repertoire diversity to recognize them and destroy the cancer cells awaits further research [24]. Once these molecules are defined, the specific T cells may be constructed and expanded to kill the cancer like the TIL adoptive therapy. Alternatively, cancer neoantigens can be used as vaccines to stimulate the T cell response in vivo.

In the literature, a significant focus has been placed on the genetic and proteomic characteristics of cancer, exploring the mutations and protein alterations that are responsible for cancer initiation and progression [99]. However, the role of the cancer microenvironment, particularly the dynamics of immune responses and the diversity of the host’s T cell population, has not been as prominently featured.

If a T cell with a cancer-specific TCR encounters a cancer cell, it can be activated and undergo clonal proliferation, rapidly producing many identical TCR-bearing cells. However, cancer micromanagement is complex [100]. Cancer can effectively target and destroy cancer cells. However, cancer can evade this response by reducing antigen presentation or creating a cancer microenvironment that suppresses the immune response. TCR species can influence the success of cancer treatment. For example, immunotherapies such as checkpoint inhibitors, which are designed to activate T cells to fight cancer, may work better in individuals with a wider variety of TCRs. Likewise, treatments that involve TILs and modifying T cells to attack cancer, such as chimeric antigen receptor (CAR)-T cell therapy (not discussed in this review) [101], depend on an understanding of the TCR in detail.

A major challenge is understanding why immunotherapy works for some patients but not others. Ongoing research aims to discover ways to enhance or alter TCR diversity to improve cancer treatment. Advanced technologies such as next-generation sequencing are used to comprehensively study TCR repertoires in cancer patients. TCR diversity research could help develop personalized medicine in cancer treatment. By examining a patient’s TCR repertoire and the antigens presented by cancer cells, treatments can be tailored to enhance immune responses against specific cancer types. Clinical trial using mRNA vaccine for melanoma [76] is an excellent example.

The intricate relationship between T cells and cancer-specific neoantigens [24] is under scrutiny to understand its depth and potential for leveraging in cancer immunotherapy. The evolving interplay between cancer metastasis [8] and the diversity of T cells [94] may vary across the different phases of cancer’s progression from an isolated cell to a group of clones, and from the formation of invasive clones to their spread to distant locations. It’s fitting to describe this deep-seated connection between cancer development and immune responses as a distinct field, termed oncolymphology, which focuses on the interactions of the immune system including lymph nodes and cancer. These interactions are vividly exemplified by the paradox presented by the SLN in the context of cancer spread and invasion. The SLN serves as the initial line of defense against cancer invasion, yet it can also become a breeding ground for cancer to grow and spread to other parts of the body [102]. Recent discoveries have shed light on this paradox, revealing that in the early stages of cancer at the primary site, chemokines released by cancer cells can reach the SLN [103] and alter its microenvironment [104]. This alteration involves attracting specific cells within the SLN to produce growth factors or cytokines, which then remodel the extracellular matrix [103] to create a pre-metastatic niche, which facilitates the invasion of cancer cells from the primary site into the lymphatic vessels, enabling them to establish a foothold in the SLN where they can thrive and multiply. Once cancer cells have securely settled in the SLN, they can further spread to systemic locations through the high endothelial venules within the SLN [105]. In addition, in an animal model, it has been shown that cancer cells colonizing the lymph node may induce cancer-immune tolerance to promote distant metastasis [106]. This study demonstrates that a tumor-specific interferon response program, established through epigenetic changes, increases the likelihood of lymph node metastasis by allowing cancer to evade natural killer cells and enhance colonization of the lymph nodes. These lymph node metastases are resistant to the cytotoxic effects of T cells, can induce regulatory T cells that are specific to the cancer antigens, and create an immune tolerance specific to the cancer. This tolerance then supports further colonization of distant sites [106]. Oncolymphology, as mentioned above, should be considered as a distinct field to describe the intricate connection between cancer development and the immune system. This topic will be expanded in a separate review article.

Spatial imaging of the cancer microenvironment and single cell analysis may open up new frontiers for determining the complex interaction of the cancer microenvironment, in particular, the immune system, and cancer growth based on their spatial relationship and the genetic characteristics of single cells. Employing single cell mapping of melanoma SLNs and T cell receptor sequencing, Yaddanapudi et al. [104] provides a novel method to study the melanoma SLN microenvironment on a detailed cellular level to suggest that the immunological changes compromise anti-melanoma immunity. Future studies using spatial imaging and single cell transcriptone analysis [107–110] will allow detailed examination of the diverse genetic and expression patterns present in each cell, offering insights into their specific functions, interactions, and contributions to the overall structure and function of tissues within the cancer microenvironment. In examining a cell’s microenvironment, spatial imaging and single-cell sequencing uncover the ways in which cells adapt to and communicate with their surroundings, including adjacent cells, the extracellular matrix, and a variety of signaling molecules. This granular perspective is essential for deciphering the complex interplay between cellular populations of the cancer microenvironment. These new techniques can differentiate the heterogeneous cell populations within a tumor and their relationships with immune and stromal cells, illuminating the pathways through which tumors grow, spread, and evade treatment. Additionally, these novel techniques can trace the developmental trajectories of cells, spotlight uncommon cell types, and follow the process of cancer proliferation. By charting the single-cell genomic and transcriptomic landscapes, researchers can discover new biomarkers and targets for treatment, paving the way for personalized cancer treatment. Novel therapeutic interventions may be developed against each step of the metastatic process at the molecular level so that metastasis may be controlled and stopped.

## Data Availability

Data sharing not applicable to this article as no datasets were generated or analyzed during the current review.
